# Triethyl­ammonium 3,4-dihy­droxy­benzoate monohydrate

**DOI:** 10.1107/S1600536810046441

**Published:** 2010-11-13

**Authors:** Li-Cai Zhu

**Affiliations:** a School of Chemistry and Environment, South China Normal University, Guangzhou 510631, People’s Republic of China

## Abstract

In the structure of the title compound, C_6_H_16_N^+^·C_7_H_5_O_4_^−^·H_2_O, O—H⋯O and N—H⋯O hydrogen bonds link the components into a three-dimensional array. The 3,4-dihy­droxy­benzoate anion is approximately planar, with a maximum deviation of 0.083 (2) Å.

## Related literature

For protocatechuic acid (3,4-dihy­droxy­benzoic acid) and its pharmacological activity, see: An *et al.* (2006 ▶); Guan *et al.* (2006 ▶); Lin *et al.* (2009 ▶); Tseng *et al.* (1998 ▶); Yip *et al.* (2006 ▶).
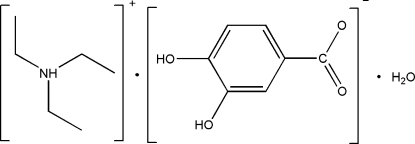


## Experimental

### 

#### Crystal data

C_6_H_16_N^+^·C_7_H_5_O_4_^−^·H_2_O
*M* *_r_* = 273.32
Orthorhombic, 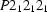

*a* = 10.7163 (16) Å
*b* = 11.5973 (17) Å
*c* = 11.7690 (17) Å
*V* = 1462.7 (4) Å^3^
*Z* = 4
Mo *K*α radiation
μ = 0.10 mm^−1^
*T* = 296 K
0.30 × 0.28 × 0.28 mm

#### Data collection

Bruker APEXII area-detector diffractometer
7531 measured reflections
1519 independent reflections
1211 reflections with *I* > 2σ(*I*)
*R*_int_ = 0.043

#### Refinement

*R*[*F* ^2^ > 2σ(*F* ^2^)] = 0.038
*wR*(*F* ^2^) = 0.093
*S* = 1.04
1519 reflections
186 parameters
3 restraints
H atoms treated by a mixture of independent and constrained refinement
Δρ_max_ = 0.20 e Å^−3^
Δρ_min_ = −0.14 e Å^−3^

### 

Data collection: *APEX2* (Bruker, 2004 ▶); cell refinement: *SAINT* (Bruker, 2004 ▶); data reduction: *SAINT*; program(s) used to solve structure: *SHELXS97* (Sheldrick, 2008 ▶); program(s) used to refine structure: *SHELXL97* (Sheldrick, 2008 ▶); molecular graphics: *SHELXTL* (Sheldrick, 2008 ▶); software used to prepare material for publication: *SHELXL97*.

## Supplementary Material

Crystal structure: contains datablocks I, global. DOI: 10.1107/S1600536810046441/zl2325sup1.cif

Structure factors: contains datablocks I. DOI: 10.1107/S1600536810046441/zl2325Isup2.hkl

Additional supplementary materials:  crystallographic information; 3D view; checkCIF report

## Figures and Tables

**Table 1:** Hydrogen-bond geometry (Å, °)

*D*—H⋯*A*	*D*—H	H⋯*A*	*D*⋯*A*	*D*—H⋯*A*
O1*W*—H2*W*⋯O2^i^	0.87 (4)	1.98 (2)	2.845 (3)	173 (4)
O1*W*—H1*W*⋯O3^ii^	0.84 (2)	2.14 (2)	2.951 (3)	162 (4)
N1—H14⋯O2^i^	0.92 (2)	1.83 (2)	2.734 (3)	166 (5)
O3—H3⋯O1^iii^	0.82	1.84	2.656 (3)	173
O4—H4*A*⋯O1^iv^	0.82	1.82	2.639 (3)	174

Symmetry codes: (i) 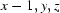; (ii) 
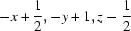
; (iii) 
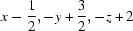
; (iv) 
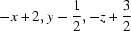
.

**Fig. 1.**
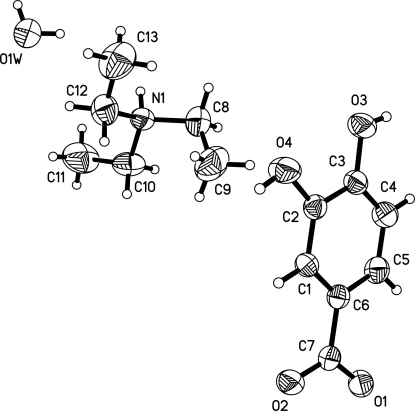
The molecular structure showing the atomic-numbering scheme and displacement ellipsoids drawn at the 50% probability level.

**Fig. 2.**
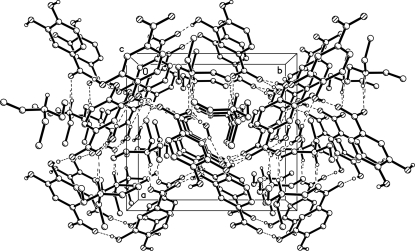
The molecular packing showing the intermolecular hydrogen bonding interactions as broken lines.

## supplementary crystallographic information

### Comment

Protocatechuic acid (3,4-dihydroxybenzoic acid) is one of the main secondary metabolites in the plant kingdom (Guan *et al.*, 2006). Significantly, it has been found that protocatechuic acid and its derivatives possess diverse pharmacological activities such as antioxidant, antiapoptosis, anticarcinogen, anticoagulatory and antiinflammatory (An *et al.*, 2006; Lin *et al.*, 2009; Tseng *et al.*, 1998; Yip *et al.*, 2006). The molecular and crystal structure of the title compound, a triethylammonium of protocatechuic acid, is presented in this article.

In the asymmetric unit of the title compound, illustrated in Fig. 1, there are a triethylammonium cation, one singly deprotonated 3,4-dihydroxybenzoate anion and one water molecule. The 3,4-dihydroxybenzoate anion is approximately planar, with a maximum deviation of any non-H atom from its plane of 0.083 (2) Å for atom O1. The orientations of the three ethyl groups of the triethylammonium cation are different. Two of the ethyl substituents are rougly in plane with the nitrogen atom and the methylene carbon atoms. The torsion angles of these two groups against the N—H bond are -53.1 for C10—C11, and -61.8 for C12—C13. The third ethyl group, C8—C9, is rotated out of this plane and is pointing downward with respect to the N—H bond with a torsion angle of 175.4°. The water molecule forms two O—H···O hydrogen bonds with two 3,4-dihydroxybenzoate anions involving O1w—H1w···O3^ii^ and O1w—H2w···O2^i^ (see Table 1 for symmetry operators and bonding geometries). The hydroxy groups of the 3,4-dihydroxybenzoate anion form O—H···O hydrogen bonds to the carboxylate groups of two adjacent anions. The N1—H14···O2^i^ hydrogen bond between the triethylammonium cation and the 3,4-dihydroxybenzoate anion is the main force influencing the orientation of the triethylammonium cation. These hydrogen bonds link the triethylammonium cations, 3,4-dihydroxybenzoate anions and water molecules into a three-dimensional array (Fig. 2).

### Experimental

A solution of triethylamine (2 mmol in 0.5 ml water) was added dropwise to a solution of protocatechuic acid (2 mmol) in acetonitrile (15 ml), and the mixture was stirred for 30 min at room temperature. After several days colourless block-like crystals, suitable for X-ray diffraction analysis, were obtained by slow evaporation of the solution.

### Refinement

H_14_ atom of the triethylammonium cation and H atoms of the water molecule were found from difference Fourier maps and refined isotropically with a restraint of N—H = 0.89 (2) Å, O—H = 0.86 (2) Å and *U*_iso_(H) = 1.5 *U*_eq_(N, O). All other H atoms were positioned geometrically and refined as riding, with O—H = 0.82 Å and C—H = 0.93, 0.96 or 0.97 Å, and with *U*_iso_(H) = 1.2 or 1.5 *U*_eq_(C, O).

### Crystal data

**Table:**

C_6_H_16_N^+^·C_7_H_5_O_4_^−^·H_2_O	*F*(000) = 592
*M**_r_* = 273.32	*D*_x_ = 1.241 Mg m^−^^3^
Orthorhombic, *P*2_1_2_1_2_1_	Mo *K*α radiation, λ = 0.71073 Å
Hall symbol: P 2ac 2ab	Cell parameters from 1465 reflections
*a* = 10.7163 (16) Å	θ = 2.5–21.3°
*b* = 11.5973 (17) Å	µ = 0.10 mm^−^^1^
*c* = 11.7690 (17) Å	*T* = 296 K
*V* = 1462.7 (4) Å^3^	Block, colourless
*Z* = 4	0.30 × 0.28 × 0.28 mm

### Data collection

**Table:**

Bruker APEXII area-detector diffractometer	1211 reflections with *I* > 2σ(*I*)
Radiation source: fine-focus sealed tube	*R*_int_ = 0.043
graphite	θ_max_ = 25.2°, θ_min_ = 2.5°
φ and ω scans	*h* = −12→6
7531 measured reflections	*k* = −13→13
1519 independent reflections	*l* = −14→14

### Refinement

**Table:**

Refinement on *F*^2^	Primary atom site location: structure-invariant direct methods
Least-squares matrix: full	Secondary atom site location: difference Fourier map
*R*[*F*^2^ > 2σ(*F*^2^)] = 0.038	Hydrogen site location: inferred from neighbouring sites
*wR*(*F*^2^) = 0.093	H atoms treated by a mixture of independent and constrained refinement
*S* = 1.04	*w* = 1/[σ^2^(*F*_o_^2^) + (0.0404*P*)^2^ + 0.2897*P*] where *P* = (*F*_o_^2^ + 2*F*_c_^2^)/3
1519 reflections	(Δ/σ)_max_ < 0.001
186 parameters	Δρ_max_ = 0.20 e Å^−^^3^
3 restraints	Δρ_min_ = −0.14 e Å^−^^3^

### Special details

**Table:**

Geometry. All e.s.d.'s (except the e.s.d. in the dihedral angle between two l.s. planes) are estimated using the full covariance matrix. The cell e.s.d.'s are taken into account individually in the estimation of e.s.d.'s in distances, angles and torsion angles; correlations between e.s.d.'s in cell parameters are only used when they are defined by crystal symmetry. An approximate (isotropic) treatment of cell e.s.d.'s is used for estimating e.s.d.'s involving l.s. planes.
Refinement. Refinement of *F*^2^ against ALL reflections. The weighted *R*-factor *wR* and goodness of fit *S* are based on *F*^2^, conventional *R*-factors *R* are based on *F*, with *F* set to zero for negative *F*^2^. The threshold expression of *F*^2^ > σ(*F*^2^) is used only for calculating *R*-factors(gt) *etc*. and is not relevant to the choice of reflections for refinement. *R*-factors based on *F*^2^ are statistically about twice as large as those based on *F*, and *R*- factors based on ALL data will be even larger.

### Fractional atomic coordinates and isotropic or equivalent isotropic displacement parameters (Å^2^)

**Table:**

	*x*	*y*	*z*	*U*_iso_*/*U*_eq_	
C1	0.9308 (3)	0.5565 (2)	0.8260 (2)	0.0342 (6)	
H1	0.9565	0.5065	0.7688	0.041*	
C6	0.9902 (3)	0.6627 (2)	0.8383 (2)	0.0325 (6)	
C3	0.7962 (3)	0.5995 (2)	0.9826 (2)	0.0368 (7)	
C2	0.8350 (3)	0.5241 (2)	0.8967 (2)	0.0344 (6)	
C5	0.9503 (3)	0.7362 (2)	0.9238 (2)	0.0414 (7)	
H5	0.9891	0.8073	0.9334	0.050*	
C4	0.8538 (3)	0.7050 (2)	0.9948 (2)	0.0422 (7)	
H4	0.8273	0.7555	1.0513	0.051*	
C7	1.0953 (3)	0.6967 (2)	0.7614 (2)	0.0359 (7)	
O1	1.1524 (2)	0.79093 (17)	0.78093 (16)	0.0458 (5)	
O2	1.12389 (19)	0.6316 (2)	0.68029 (18)	0.0531 (6)	
O4	0.7734 (2)	0.42081 (17)	0.89033 (19)	0.0537 (6)	
H4A	0.8011	0.3831	0.8370	0.081*	
O3	0.7002 (2)	0.56347 (19)	1.05036 (17)	0.0491 (6)	
H3	0.6853	0.6129	1.0983	0.074*	
N1	0.3697 (2)	0.6142 (2)	0.6203 (2)	0.0417 (6)	
C10	0.4121 (3)	0.6946 (3)	0.5286 (3)	0.0608 (9)	
H10A	0.4949	0.6720	0.5039	0.073*	
H10B	0.4178	0.7720	0.5595	0.073*	
C12	0.3732 (4)	0.4905 (3)	0.5826 (3)	0.0574 (9)	
H12A	0.3205	0.4817	0.5162	0.069*	
H12B	0.4578	0.4710	0.5608	0.069*	
C11	0.3266 (4)	0.6960 (4)	0.4277 (3)	0.0885 (14)	
H11A	0.3283	0.6220	0.3912	0.133*	
H11B	0.3536	0.7541	0.3751	0.133*	
H11C	0.2431	0.7128	0.4523	0.133*	
C13	0.3304 (5)	0.4082 (3)	0.6726 (4)	0.0840 (13)	
H13A	0.3883	0.4094	0.7348	0.126*	
H13B	0.3264	0.3318	0.6415	0.126*	
H13C	0.2493	0.4307	0.6990	0.126*	
C8	0.4404 (3)	0.6357 (3)	0.7284 (3)	0.0577 (9)	
H8A	0.4001	0.5934	0.7894	0.069*	
H8B	0.4349	0.7171	0.7466	0.069*	
C9	0.5759 (3)	0.6018 (4)	0.7247 (4)	0.0822 (13)	
H9A	0.5825	0.5197	0.7161	0.123*	
H9B	0.6159	0.6249	0.7941	0.123*	
H9C	0.6157	0.6392	0.6616	0.123*	
H14	0.288 (2)	0.633 (4)	0.637 (4)	0.123*	
O1W	0.0402 (3)	0.5325 (3)	0.4727 (2)	0.0719 (8)	
H1W	−0.035 (2)	0.514 (4)	0.482 (4)	0.108*	
H2W	0.066 (4)	0.568 (3)	0.533 (3)	0.108*	

### Atomic displacement parameters (Å^2^)

**Table:**

	*U*^11^	*U*^22^	*U*^33^	*U*^12^	*U*^13^	*U*^23^
C1	0.0341 (15)	0.0357 (15)	0.0328 (14)	0.0014 (13)	0.0032 (13)	−0.0061 (12)
C6	0.0319 (15)	0.0328 (15)	0.0329 (14)	0.0021 (12)	−0.0029 (12)	0.0010 (12)
C3	0.0340 (16)	0.0435 (17)	0.0327 (15)	0.0026 (14)	0.0009 (13)	−0.0001 (13)
C2	0.0343 (16)	0.0341 (14)	0.0347 (14)	−0.0005 (12)	−0.0006 (13)	−0.0044 (13)
C5	0.0458 (19)	0.0339 (15)	0.0446 (16)	−0.0034 (14)	−0.0005 (15)	−0.0077 (14)
C4	0.0415 (17)	0.0403 (18)	0.0448 (17)	0.0033 (15)	0.0063 (15)	−0.0131 (14)
C7	0.0336 (16)	0.0405 (17)	0.0335 (15)	−0.0009 (14)	−0.0043 (12)	−0.0001 (13)
O1	0.0550 (13)	0.0438 (12)	0.0387 (11)	−0.0165 (11)	0.0005 (10)	0.0018 (9)
O2	0.0471 (14)	0.0624 (14)	0.0497 (12)	−0.0131 (11)	0.0134 (11)	−0.0191 (11)
O4	0.0581 (15)	0.0441 (13)	0.0590 (15)	−0.0146 (11)	0.0204 (12)	−0.0139 (11)
O3	0.0463 (14)	0.0550 (13)	0.0459 (12)	−0.0037 (11)	0.0149 (10)	−0.0132 (10)
N1	0.0393 (15)	0.0445 (14)	0.0413 (14)	−0.0017 (12)	0.0058 (12)	−0.0020 (11)
C10	0.055 (2)	0.061 (2)	0.067 (2)	−0.0040 (19)	0.0120 (18)	0.0178 (18)
C12	0.065 (2)	0.0475 (19)	0.060 (2)	0.0024 (17)	0.0031 (19)	−0.0145 (17)
C11	0.081 (3)	0.117 (4)	0.068 (3)	0.002 (3)	−0.003 (2)	0.039 (3)
C13	0.098 (3)	0.055 (2)	0.099 (3)	−0.013 (2)	−0.013 (3)	0.013 (2)
C8	0.060 (2)	0.060 (2)	0.0527 (19)	−0.0054 (19)	−0.0044 (18)	−0.0096 (17)
C9	0.055 (2)	0.099 (3)	0.094 (3)	−0.003 (2)	−0.018 (2)	0.003 (3)
O1W	0.0682 (19)	0.0815 (19)	0.0658 (16)	−0.0147 (16)	0.0034 (15)	−0.0133 (14)

### Geometric parameters (Å, °)

**Table:**

C1—C2	1.374 (4)	C10—C11	1.500 (5)
C1—C6	1.395 (4)	C10—H10A	0.9700
C1—H1	0.9300	C10—H10B	0.9700
C6—C5	1.387 (4)	C12—C13	1.497 (5)
C6—C7	1.498 (4)	C12—H12A	0.9700
C3—O3	1.368 (3)	C12—H12B	0.9700
C3—C4	1.378 (4)	C11—H11A	0.9600
C3—C2	1.400 (4)	C11—H11B	0.9600
C2—O4	1.370 (3)	C11—H11C	0.9600
C5—C4	1.379 (4)	C13—H13A	0.9600
C5—H5	0.9300	C13—H13B	0.9600
C4—H4	0.9300	C13—H13C	0.9600
C7—O2	1.255 (3)	C8—C9	1.505 (5)
C7—O1	1.273 (3)	C8—H8A	0.9700
O4—H4A	0.8200	C8—H8B	0.9700
O3—H3	0.8200	C9—H9A	0.9600
N1—C10	1.497 (4)	C9—H9B	0.9600
N1—C12	1.501 (4)	C9—H9C	0.9600
N1—C8	1.502 (4)	O1W—H1W	0.841 (19)
N1—H14	0.92 (2)	O1W—H2W	0.87 (4)
			
C2—C1—C6	121.3 (3)	C11—C10—H10B	109.0
C2—C1—H1	119.3	H10A—C10—H10B	107.8
C6—C1—H1	119.3	C13—C12—N1	113.1 (3)
C5—C6—C1	118.6 (3)	C13—C12—H12A	108.9
C5—C6—C7	120.6 (2)	N1—C12—H12A	108.9
C1—C6—C7	120.9 (2)	C13—C12—H12B	108.9
O3—C3—C4	123.2 (3)	N1—C12—H12B	108.9
O3—C3—C2	117.0 (3)	H12A—C12—H12B	107.8
C4—C3—C2	119.8 (3)	C10—C11—H11A	109.5
O4—C2—C1	124.5 (2)	C10—C11—H11B	109.5
O4—C2—C3	116.3 (2)	H11A—C11—H11B	109.5
C1—C2—C3	119.3 (3)	C10—C11—H11C	109.5
C4—C5—C6	120.7 (3)	H11A—C11—H11C	109.5
C4—C5—H5	119.7	H11B—C11—H11C	109.5
C6—C5—H5	119.7	C12—C13—H13A	109.5
C3—C4—C5	120.4 (3)	C12—C13—H13B	109.5
C3—C4—H4	119.8	H13A—C13—H13B	109.5
C5—C4—H4	119.8	C12—C13—H13C	109.5
O2—C7—O1	122.5 (3)	H13A—C13—H13C	109.5
O2—C7—C6	119.0 (2)	H13B—C13—H13C	109.5
O1—C7—C6	118.6 (2)	N1—C8—C9	114.8 (3)
C2—O4—H4A	109.5	N1—C8—H8A	108.6
C3—O3—H3	109.5	C9—C8—H8A	108.6
C10—N1—C12	112.0 (2)	N1—C8—H8B	108.6
C10—N1—C8	110.7 (3)	C9—C8—H8B	108.6
C12—N1—C8	113.3 (3)	H8A—C8—H8B	107.6
C10—N1—H14	107 (3)	C8—C9—H9A	109.5
C12—N1—H14	108 (3)	C8—C9—H9B	109.5
C8—N1—H14	105 (3)	H9A—C9—H9B	109.5
N1—C10—C11	113.0 (3)	C8—C9—H9C	109.5
N1—C10—H10A	109.0	H9A—C9—H9C	109.5
C11—C10—H10A	109.0	H9B—C9—H9C	109.5
N1—C10—H10B	109.0	H1W—O1W—H2W	108 (4)

### Hydrogen-bond geometry (Å, °)

**Table:**

*D*—H···*A*	*D*—H	H···*A*	*D*···*A*	*D*—H···*A*
O1W—H2W···O2^i^	0.87 (4)	1.98 (2)	2.845 (3)	173 (4)
O1W—H1W···O3^ii^	0.84 (2)	2.14 (2)	2.951 (3)	162 (4)
N1—H14···O2^i^	0.92 (2)	1.83 (2)	2.734 (3)	166 (5)
O3—H3···O1^iii^	0.82	1.84	2.656 (3)	173
O4—H4A···O1^iv^	0.82	1.82	2.639 (3)	174

Symmetry codes: (i) *x*−1, *y*, *z*; (ii) −*x*+1/2, −*y*+1, *z*−1/2; (iii) *x*−1/2, −*y*+3/2, −*z*+2; (iv) −*x*+2, *y*−1/2, −*z*+3/2.
